# Combined inflammatory parameters and tertiary lymphoid structure predict prognosis in patients with resectable non-small cell lung cancer treated with neoadjuvant chemoimmunotherapy

**DOI:** 10.3389/fimmu.2023.1244256

**Published:** 2023-12-14

**Authors:** Fuhao Xu, He Zhu, Yinjun Dong, Li Li, Ning Liu, Shuanghu Yuan

**Affiliations:** ^1^ Department of Radiation Oncology, Shandong Cancer Hospital and Institute, Shandong First Medical University and Shandong Academy of Medical Sciences, Shandong Cancer Hospital Affiliated to Shandong First Medical University, Jinan, China; ^2^ Department of Thoracic Surgery, Shandong Cancer Hospital and Institute Shandong First Medical University and Shandong Academy of Medical Sciences, Jinan, China

**Keywords:** inflammatory parameter, tertiary lymphoid structure, tumor immune microenvironment, immunotherapy, non-small cell lung cancer, prognosis

## Abstract

**Introduction:**

Neoadjuvant chemoimmunotherapy shows great potential for patients with non-small cell lung cancer (NSCLC), but no clear prognostic markers have been identified. This study investigates the correlation between inflammatory parameters and the expression of tertiary lymphoid structures (TLS) and the predictive ability of inflammatory parameters combined with TLS for disease-free survival (DFS) in patients with resectable NSCLC receiving neoadjuvant chemotherapy.

**Materials and methods:**

We retrospectively analyzed the clinical data and hematological parameters of 117 patients with NSCLC who underwent neoadjuvant chemoimmunotherapy and radical surgery. TLS were evaluated by observing H&E stained and immunohistochemically stained tissue sections. Univariate chi-square and multifactor logistic analyses were used to determine the correlation between hematological parameters and TLS. The Kaplan–Meier method, univariate and multivariate Cox regression analysis and constructed nomogram models were used to assess the prognostic value of the investigated parameters on DFS. Receiver operating characteristic (ROC) curves analyses were used to compare the performances of the three models.

**Results:**

After logistic analysis, it was found that platelet-to-lymphocyte ratio (PLR) ≤288.78 (odds ratio OR=0.122, P=0.009) was an independent predictor of high TLS expression. The Cox regression analyses showed that Histology (HR=0.205, P=0.002), systemic immune inflammation index (SII) (HR=2.758, P=0.042) and TLS (HR=0.057, P<0.05) were independent prognostic factors in patients with NSCLC. The combined SII-TLS model was better than the single-indicator model in assessing the 1-year and 18-months DFS rates in patients with NSCLC.

**Conclusion:**

Our study showed that PLR was an independent predictor of TLS and that both TLS and SII predicted prognosis in patients with neoadjuvant chemoimmunotherapy-resectable NSCLC; however, combining SII and TLS to assess DFS was more accurate than using either parameter alone.

## Introduction

Lung cancer is a global health problem. Approximately 80-85% of lung cancer cases are non-small cell lung cancers (NSCLCs). The latest global cancer statistics indicate that over two million people are newly diagnosed with lung cancer each year. Moreover, recently, lung cancer has become one of the leading causes of cancer-related deaths worldwide (1, 2). Tumor immunotherapy has become one of the most successful approaches in cancer treatment in recent years (3). Immunotherapies for tumors, such as immune checkpoint blockade (ICB), can relieve tumor tolerance to immunity and allow immune cells to recognize and kill the tumor cells. Several ICBs currently undergoing basic and clinical research. Among them, inhibitors targeting the immune checkpoint programmed cell death protein 1 (PD-1) molecule are at the forefront of immunotherapy (4).

The tumor immune microenvironment refers to the microenvironment surrounding a tumor and consists tumor, immune, and mesenchymal cells. Numerous studies have shown that the tumor immune microenvironment has a crucial role in immunotherapy (5). Tertiary lymphoid structures (TLSs) are structured aggregates of immune cells that form in non-lymphoid tissues after birth and are important components of the tumor immune microenvironment (6). TLSs are usually associated with a positive prognosis in most cancer types, and their prognostic value is independent of TNM stage. TLSs have also been shown to be associated with tumor development and progression in certain cancer types (6). A recent study examined on-treatment tumor samples and discovered that ICB treatment stimulates the development of TLSs and that TLSs contribute to the clinical response to immune checkpoint blocking. After neoadjuvant immunotherapy for melanoma and uroepithelial carcinoma, patients who showed immunotherapeutic effects had higher numbers of TLS-associated immune cells in their tumors than before (7). In addition, an increase in the number of TLSs was observed in lesions after neoadjuvant PD-1 blocker treatment for NSCLC (8).

TLSs form in tissues in response to inflammation, require a continuous inflammatory environment to be maintained, and share some structural similarities with lymph nodes; however, TLSs lack a surrounding sealing structure. This structural characteristic may allow cellular components to permeate the surrounding tissues directly, increasing the possibility that immune cells will be affected by components of the inflammatory environment (9). However, not all inflammatory stimuli drive the formation of TLSs, which may depend on specific microenvironmental components. The Systemic Immune Inflammatory Index (SII) is based on a prognostic score of inflammation and immunity, which is calculated as platelet count*neutrophil count/lymphocyte count. The SII, neutrophil-to-lymphocyte ratio (NLR), lymphocyte-to-monocyte ratio (LMR), and platelet-to-lymphocyte ratio (PLR), which reflect the balance between inflammatory factors and immunity in the body, have been shown to be effective in predicting patient prognosis (10–12). This finding indicates that the state of the tumor inflammatory microenvironment may be closely linked to the antitumor immune response and has an important impact on the prognosis of patients with NSCLC.

Despite effective surgical treatment, a proportion of patients with resectable stage IB–IIIA NSCLC still have poor prognosis due to recurrence. Neoadjuvant chemotherapy has shown substantial potential in this group of patients. The use of 2-4 cycles of neoadjuvant immunotherapy combined with platinum-based and paclitaxel-liked chemotherapy before surgery can improve long-term survival, reduce tumor stage, improve resection rates, and provide timely management of micrometastases. Results of the CheckMate-816 phase III trial showed a significant improvement in 3-year disease-free survival with 2-4 cycles of preoperative Nivolumab in combination with chemotherapy compared to surgery alone (63% vs 50%) (13). However, to date, no clear predictive prognostic markers for neoadjuvant chemoimmunotherapy have been identified.

Therefore, this study investigated the effect of hematological parameters (SII, PLR, NLR, and LMR), which represent the inflammatory state of the tumor microenvironment, on TLSs and compared the predictive ability of these hematological parameters, TLSs, and hematological parameters combined with that of TLS alone on disease-free survival (DFS) in patients with NSCLC treated with neoadjuvant chemoimmunotherapy. Notably, our results provide an accurate model for predicting the prognosis of patients with NSCLC treated with neoadjuvant chemoimmunotherapy.

## Materials and methods

### Patients and samples

**Figure 1:**
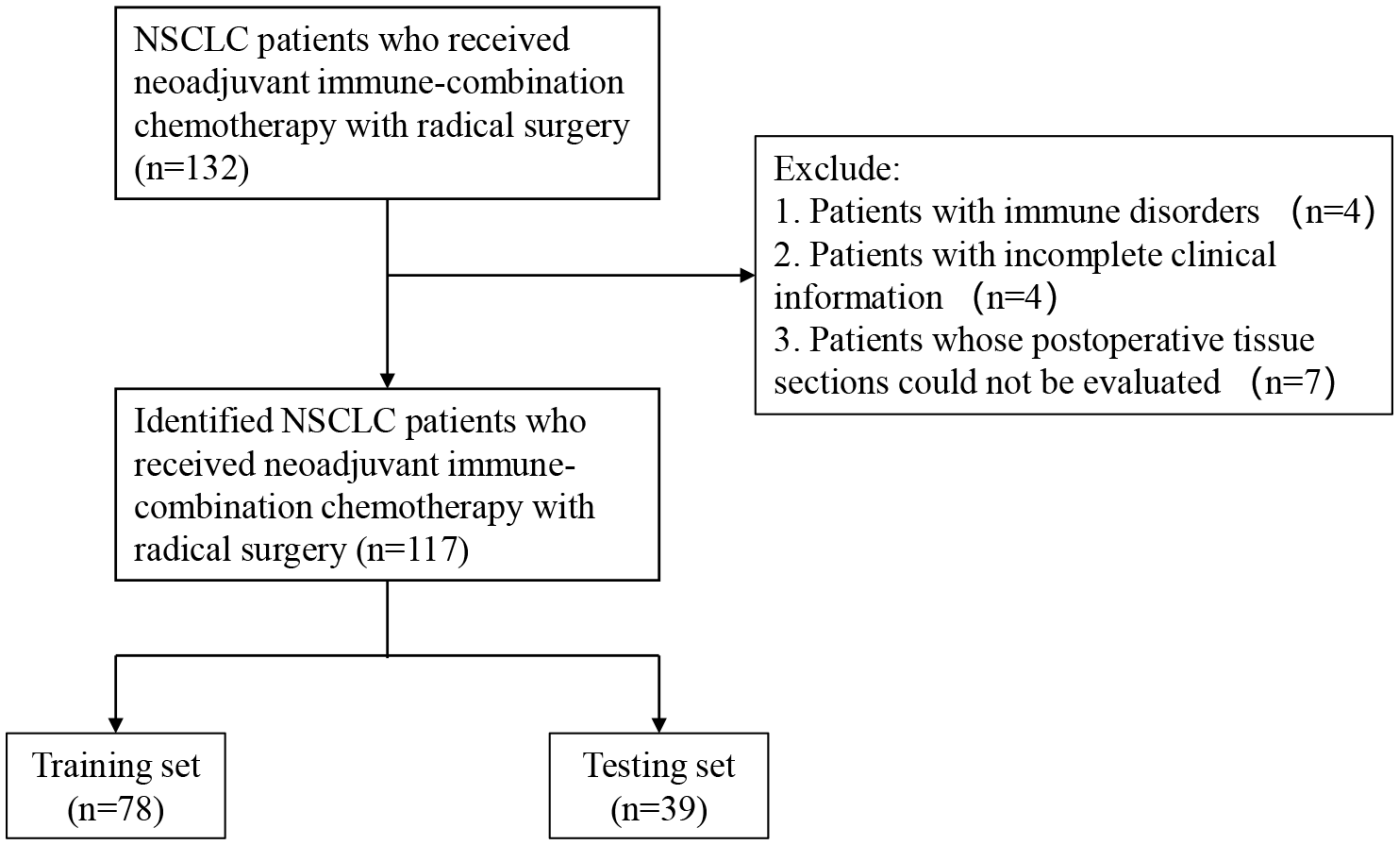
Flow chart for the inclusion and exclusion of NSCLC patients and analysis of tumor information. NSCLC, non-small-cell lung cancer.

We retrospectively evaluated 117 patients with NSCLC diagnosed *via* surgical pathology at the Shandong Cancer Hospital between January 2020 and June 2022. All patients were driver gene negative and had not received targeted therapy. All patients underwent radical surgery and received at least two cycles of preoperative neoadjuvant therapy. Specific medications: PD-1 ICBs (Pembrolizumab, Sintilimab, Tislelizumab, Camrelizumab) combined with platinum-based drugs (Cisplatin, Carboplatin) and paclitaxel drugs (nab-paclitaxel) or pemetrexed disodium. Administered intravenously on day 1 of a 21-day cycle. R0 resection was obtained in all patients. For early-stage patients, we performed regular follow-up after surgery. For patients with advanced stage and high risk of recurrence factors, we performed immune maintenance therapy after surgery (limited to PD-L1 TC ≥1%). Patients with active autoimmune disease; a previous history of autoimmune disease; or congenital or acquired immune deficiency, such as human immunodeficiency virus infection, active hepatitis B, or hepatitis C, were excluded from the study. The included patients underwent regular follow-up at the outpatient clinic every 3–6 months for 2 years after surgery and annually thereafter. The time of postoperative pathological diagnosis was used as the starting point for observation, and each patient was followed up periodically by outpatient review and telephone until disease progression or death, with a final follow-up date of May 22, 2023. Written informed consents were provided by all participants. It was carried out in accordance with the Helsinki Declaration and approved by the Ethics Review Committee of Shandong Cancer Hospital in China. (SDTHEC2022010006). The process of patient screening is shown in **Figure 1**.

### Data collection

Tissue wax blocks, general clinical data, postoperative pathological data, and laboratory test results, such as routine blood within one week before neoadjuvant immune combination chemotherapy, were collected from patients with NSCLC who met the enrollment criteria by reviewing medical records. The primary data included patient sex, age, type of pathology, TNM stage, neutrophil, monocyte, lymphocyte, and platelet counts. On the basis of the eighth edition of the AJCC cancer staging system, NSCLCs were staged. The tissue wax blocks used in this study were obtained from the Department of Pathology at Shandong Cancer Hospital.

### Histopathological analysis

Two or three sections were prepared from each wax block. Sections were stained using hematoxylin and eosin (HE), and the abundance of TLSs in the tumor tissue was scored as 0, 1, or 2: (a) a score of 0 indicates no TLS; (b) a score of 1 indicates that the tumor has less than three TLSs; and (c) a score of 2 indicates that the tumor has at least three TLSs. Based on previous studies, we defined TLSs with active germinal centers (GC) as fully mature secondary follicle-like TLSs (14). We defined patients with an abundance score of 2 and mature TLS as TLS(+) or otherwise as TLS (–). We evaluated the TLSs to an extent of 3 mm at the intertumoral and peritumoral locations. Sections were stained with anti-human antibodies against *CD3*, *CD20*, and *CD21* to validate the presence of TLS through T, B, and follicular dendritic cells. TLSs scores were independently assessed by two senior pathologists under double-blind conditions for outcome assessment, and when the pathologist scores differed, the higher score was used.

### Statistical analysis

DFS was calculated as the time between postoperative pathological diagnosis and disease progression or death due to NSCLC. Major pathologic response (MPR) was defined as the presence of ≤10% residual tumor cells in the surgical resection specimen, while the non-MPR group included patients who did not achieve MPR. Statistical analyses were performed using RStudio and SPSS software version 25.0. Receiver operating characteristic (ROC) curves and Jamovi statistical software version 2.3.21 were used to determine the optimal cutoff values for blood parameters (SII, PLR, LMR, and NLR). Chi-square and multifactorial binary logistic analyses were used to determine the correlation between blood parameters and TLSs, and Cox proportional hazards regression analysis was used to determine the indicators associated with DFS. DFS was compared using the Kaplan–Meier method, and survival curves were compared using the log-rank test. Differences with p values <0.05 were considered statistically significant. Based on the independent predictors identified, the patients were divided into training and validation sets at a ratio of 2:1. Furthermore, using R language statistical software, nomogram models were constructed to predict DFS based on SII, TLS, and SII combined with TLS. In addition, ROC curves analyses were used to compare the performances of the three models.

## Results

### Patient characteristics

**Figure 2:**
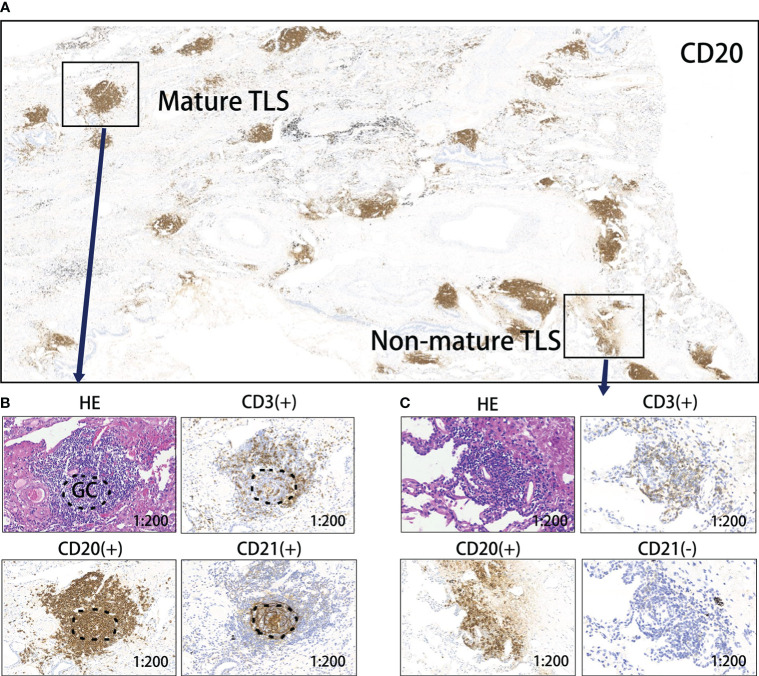
Levels of TLS maturity. **(A)** Representative image of CD20 staining of TLSs in a responder after neoadjuvant treatment. **(B)** Mature TLS, dense lymphocytic aggregates (CD3+, CD20+) with follicular dendritic cells (CD21+); **(C)** Immature TLS, dense lymphocytic aggregates (CD3+, CD20+) without follicular dendritic cells (CD21-). TLS, tertiary lymphoid structure; GC, germinal center.

A total of 117 patients with NSCLC who received neoadjuvant chemo-immunotherapy and underwent radical surgery, were included in the study (96(82.1%) male). The median age at diagnosis was 63 (44–102) years, and the median follow-up duration was 15.5 months. Additionally, 71 (60.7%) patients had squamous cell carcinoma and 46 (39.3%) had adenocarcinoma. Among all patients, 14 (12.0%), 38 (32.5%), and 65 (55.5%) patients scored 0, 1, and 2, respectively, for TLS abundance. By observation, 77 (65.8%) patients had mature TLSs; maturation states are shown in **Figure 2**. Fifty-eight (49.6%) patients had TLS (+). Other clinical characteristics are shown in **Supplementary Table 1**.

### Systemic inflammation parameters with TLS

The ROC curve analysis was used to determine the optimal cutoff values for the SII, NLR, PLR, and LMR before treatment. This value was used as the threshold value to group inflammatory indicators (**Supplementary Table 2**). The TLS (+) and TLS (–) subgroups were compared according to cut-off values to investigate the relationship between inflammatory parameters and TLSs. The univariate chi-square analysis indicated that the NLR (χ2 = 7.873, P=0.005), and PLR (χ2 = 13.835, P<0.001) significantly correlated with the TLS expression profile. Further inclusion of NLR and PLR in a multifactorial binary logistic analysis revealed that PLR (OR=0.122, 95% CI=0.025-0.592, P=0.009) was an independent predictor of TLS expression level, PLR ≤ 288.78 was considered a protective factor for high TLS expression; lower PLR suggested high TLS expression level (**Table 1**).

**Table 1:** Univariate and multivariate logistic analyses on inflammatory parameters and the expression profile of TLSs.

Variable	TLS+ (n=58)	TLS-(n=59)	Univariate analysis		Multivariate analysis	
			χ2	*P*	*P*	*OR (95%CI)*
**SII**			2.681	0.102		
≤822.63	40	32				
>822.63	18	27				
**NLR**			7.873	0.005	0.168	0.543(0.228-1.292)
≤3.59	44	30				
>3.59	14	29				
**PLR**			13.835	<0.001	0.009	0.122(0.025-0.592)
≤288.78	56	42				
>288.78	2	17				
**LMR**			1.027	0.311		
≤3.04	30	25				
>3.04	28	34				

Statistical significance was set at P < 0.05. The expected count should be <5 to follow the Fisher’s exact test results CI, confidence interval; LMR, lymphocyte-to-monocyte ratio; OR, odds ratio; PLR, platelet-to-lymphocyte ratio; NLR, neutrophil-to-lymphocyte ratio; SII, Systemic Inflammatory Index; TLS, tertiary lymphoid structures.

### Association between tertiary lymphoid structures and neoadjuvant therapy efficacy and prognosis in NSCLC

**Figure 3:**
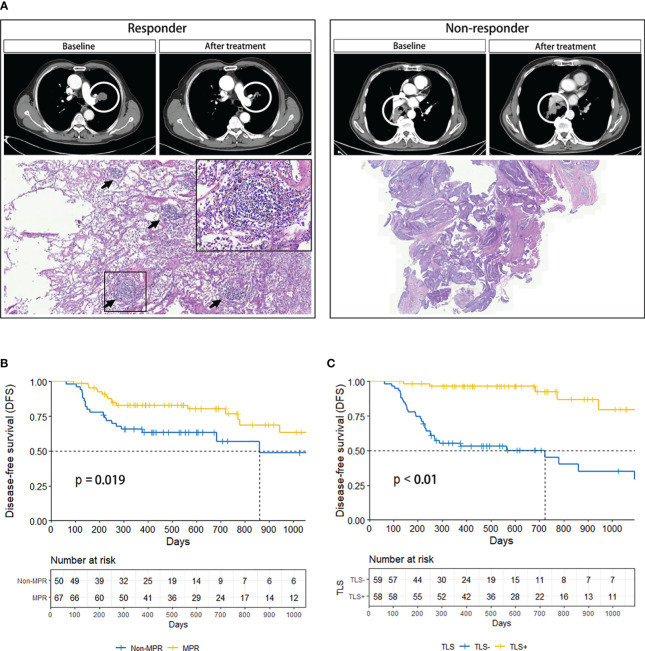
**(A)** Comparison of CT and pathology images of responder (left) and non-responder (right). The black arrow marks the TLSs. **(B)** DFS curves of MPR and Non-MPR in NSCLC patients. **(C)** DFS curves of TLS+ and TLS- in NSCLC patients. CT, computed tomography; DFS, disease-free survival; MPR, major pathologic response; NSCLC, non-small-cell lung cancer; TLS, tertiary lymphoid structure.

TLSs were more readily observed in patients responsive to neoadjuvant immune-chemotherapy; however, were rarely found in non-responders (**Figure 3A**). Based on the pathological response after treatment, patients with NSCLC were divided into MPR and non-MPR groups. Kaplan-Meier survival curves showed significantly improved DFS in the MPR group compared to the non-MPR group (P=0.019), demonstrating an association between tumor regression induced by neoadjuvant therapy and DFS (**Figure 3B**). Additionally, the TLS (+) group demonstrated markedly improved DFS compared to the TLS (–) group (P<0.01), validating the advantageous prognostic value of TLSs in NSCLC patients (**Figure 3C**).

### Prognostic survival analysis combining systemic inflammation parameters with TLS

**Figure 4:**
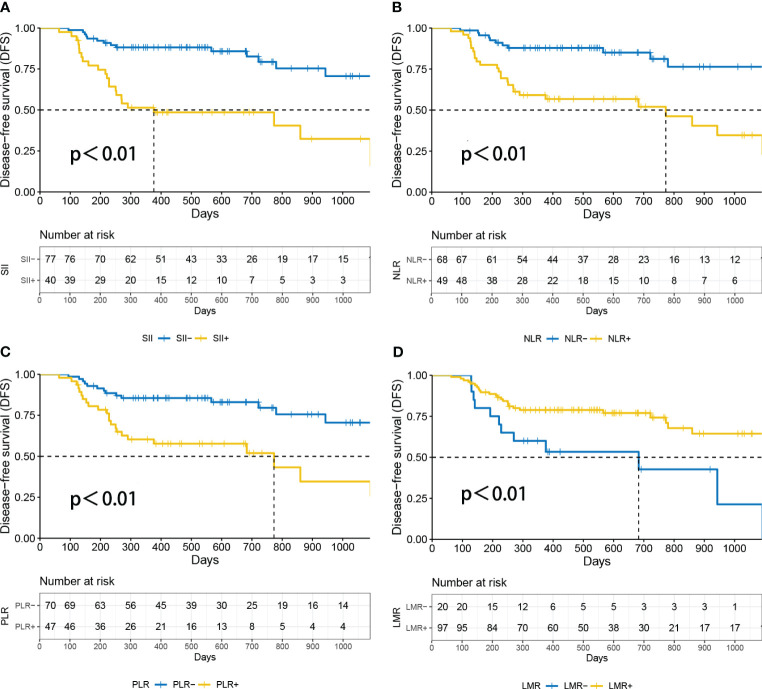
Survival analysis according to patients’ inflammatory parameters. *P*<0.05 was considered statistically significant. **(A)** DFS curves of SII^high^ and SII^low^ in NSCLC patients. **(B)** Disease-free survival (DFS) curves of NLR^high^ and NLR^low^ in NSCLC patients. **(C)** DFS curves of PLR^high^ and PLR^low^ in NSCLC patients. **(D)** Disease-free survival (DFS) curves of LMR^high^ and LMR^low^ in NSCLC patients. DFS, disease-free survival; LMR, lymphocyte-to-monocyte ratio; NLR, neutrophil-to-lymphocyte ratio; PLR, platelet-to-lymphocyte ratio; SII, Systemic Inflammatory Index.

Based on the DFS durations, the optimal cutoff values for inflammatory parameters in patients with NSCLC were determined (SII=1014, PLR=175, LMR=2.22, NLR=3.21) (15). As a result, patients were divided into groups with high and low inflammatory parameters according to the optimal cut-off values. A statistically significant difference in survival between the high and low inflammatory parameters groups was found using Kaplan–Meier survival curves for SII (P<0.01), NLR (P<0.01), PLR (P<0.01) and LMR (P<0.01) groups (**Figure 4**). In the univariate Cox regression analysis of NSCLC, age, sex, smoking history, T stage, N stage, histology, systemic inflammation parameters, and TLS expression were included. Age, histology, T stage, NLR, PLR, SII, LMR and TLS affected DFS (**Table 2**). A multivariate Cox regression analysis was subsequently performed; histology, (HR=0.205, P=0.002), SII (HR=2.758, P=0.042) and TLS (HR=0.057, P<0.05) were independent prognostic factors for patients with NSCLC receiving neoadjuvant chemo-immunotherapy (**Table 2**).

**Table 2:** Univariate and multivariate COX analysis for factors associated with DFS in NSCLC patients with neoadjuvant chemo-immunotherapy.

Variable	Univariate analysis			Multivariate analysis		
	HR	95%CI	*P* value	HR	95%CI	*P* value
**Age**	1.035	1.004-1.006	0.024	1.029	0.991-1.069	0.134
**Sex (vs female)**	1.960	0.814-4.723	0.134			
**Smoking history (vs never smoker)**	1.541	0.789-3.012	0.206			
**Histology (vs adenocarcinoma)**	0.255	0.113-0.574	0.001	0.205	0.075-0.559	0.002
**T Stage (vs 1)**			0.023			0.064
2	3.128	0.418-23.387	0.267	1.866	0.217-16.021	0.569
3	8.251	1.063-64.073	0.044	6.032	0.638-57.015	0.117
4	3.630	0.417-31.589	0.243	1.857	0.179-19.220	0.604
**N Stage (vs 0)**			0.362			
1	0.944	0.347-2.568	0.910			
2	1.011	0.429-2.382	0.981			
3	6.202	0.744-51.727	0.092			
**NLR^a^**	3.322	1.726-6.393	<0.05	1.937	0.709-5.291	0.198
**PLR^b^**	3.056	1.614-5.786	0.001	1.866	0.760-4.579	0.173
**LMR^c^**	0.356	0.178-0.710	0.003	1.056	0.428-2.601	0.906
**SII^d^**	4.818	2.470-9.398	<0.05	2.758	1.039-7.319	0.042
**TLS^e^**	0.130	0.054-0.311	<0.05	0.057	0.018-0.182	<0.05

Statistical significance was set at P < 0.05. a.Divided into NLR^High^ and NLR^Low^. b.Divided into PLR^High^ and PLR^Low^. c.Divided into LMR^High^ and LMR^Low^. d.Divided into SII^High^ and SII^Low^. e.Divided into TLS+ and TLS-.

CI, confidence interval; DFS, disease free survival; HR, hazards ratio; LMR, lymphocyte-to-monocyte ratio; OR, odds ratio; PLR, platelet-to-lymphocyte ratio; NLR, neutrophil-to-lymphocyte ratio; NSCLC, non-small-cell lung cancer; SII, Systemic Inflammatory Index; TLS, tertiary lymphoid structures.

### Comparison of nomogram prognostic models

**Figure 5:**
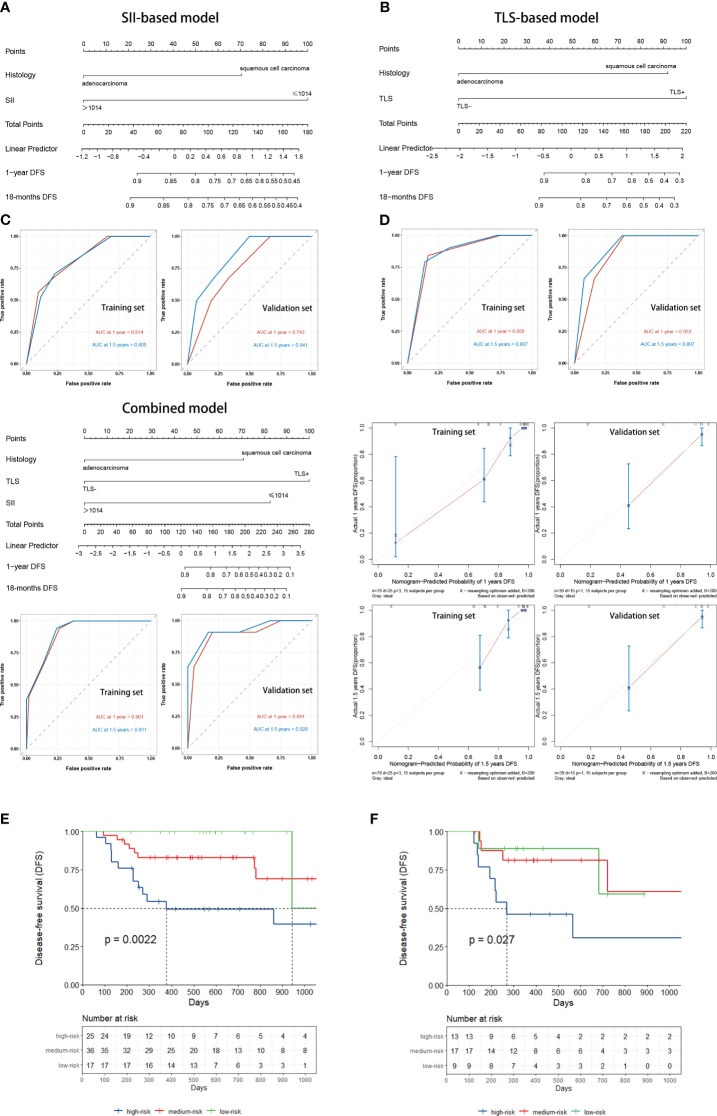
**(A)** SII-based model and ROC curves to predict the DFS of NSCLC patients receiving neoadjuvant chemoimmunotherapy. **(B)** TLSs-based model and ROC curves to predict DFS. **(C)** Combined model and ROC curves to predict DFS. **(D)** The calibration curve was close to ideal. **(E)** Kaplan–Meier curve using the tertile of the model-predicted score (Training set). Patients were grouped into low-, medium-, and high-risk groups. **(F)** Kaplan–Meier curve using the tertile of the model-predicted score (Validation set) with patients grouped into low-, medium-, and high-risk groups. DFS, disease-free survival; NSCLC, non-small-cell lung cancer; ROC, receiver operating characteristic; SII, Systemic Inflammatory Index; TLS, tertiary lymphoid structure.

The training set of the combined model included 78 patients, while the validation set included 39 patients. There were no significant between-group differences in variables. The validation set comprised 26 (66.7%) patients with squamous cell carcinoma and 13 (33.3%) with adenocarcinoma. Eighteen (49.2%) patients in the validation set tested positive for TLS, while 11 (28.2%) tested positive for SII. The performance of three constructed nomogram models SII (**Figure 5A**), TLS (**Figure 5B**), and SII combined with TLS (**Figure 5C**) was evaluated; the results are displayed in **Table 3**. The calibration curve of the combined model was close to ideal curve (**Figure 5D**). A comparison of the area under the ROC curve of the three models revealed that for the 1-year DFS prediction, the combined model had a significantly improved performance compared to the SII- and TLS-based model. Similar results were obtained for the predictive model comparison of the 18-months DFS durations (**Table 3** **).** In addition, we perform a validation of the combined model using an independent validation cohort. According to the tertile of the model predicted score, patients were organized into low-, medium-, and high-risk groups. The validation set was stratified by risk scores derived from the training data. Survival curves are presented in **Figures 5E, F**.

**Table 3:** Comparison of Nomogram Models.

End point	Models	AUC	
		Training cohort	Validation cohort
1-year DFS	Combined model	0.901	0.891
	SII-based model	0.814	0.743
	TLS-based model	0.858	0.853
18-months DFS	Combined model	0.911	0.928
	SII-based model	0.805	0.841
	TLS-based model	0.867	0.897

Combined model: a combined nomogram model based on the expression of TLSs with SII

AUC, area under curve; DFS, disease-free survival; SII, Systemic Inflammatory Index; TLS, tertiary lymphoid sructure.

## Discussion

In this study, we assessed the immune infiltration status of patients with neoadjuvant chemoimmunotherapy-resectable NSCLC using pretreated peripheral blood and TLSs. Our results indicated that both high expression levels of TLS and low levels of SII were associated with a positive prognosis in patients with NSCLC patients, however, limitations of the single-parameter-based models were observed when predicting the DFS duration of the patients. Therefore, we combined these two factors with basic patient clinical information to design a prognostic model from a more comprehensive perspective. The resulting model was observed to better predict the DFS of patients with NSCLC and thus, will help clinicians treat patients more effectively.

In recent years, because of the positive predictive performance of TLS in melanoma (16), esophageal cancer (17), lung cancer (18), liver cancer (19) and other tumors, the concept of TLS has gradually become well known to researchers and clinicians and has become a popular prognostic biomarker independent of TNM staging. TLSs are immune cells capable of an anti-tumor immune response (TFH cells, follicular B cells, DC-LAMP+ mature dendritic cells, etc.), and their formation indicates the presence of continuous anti-tumor T and B cell immune responses at the tumor site; thus, their abundance and maturity can reflect the immune infiltration status of patients with NSCLC (20). ICB has been found to increase the abundance of TLSs in several cancer types (7, 21, 22). Therefore, this study was conducted to assess the abundance and maturation of TLSs in patients with NSCLC after immunotherapy, which could reflect the immune status of patients more accurately. These results were consistent with those of the majority of studies, where a higher abundance and maturity of TLSs represented patients with a positive prognosis. It has also been hypothesized that the location of the TLSs affects their prognostic value. A previous study found that in hepatocellular carcinoma, the presence of peritumoral TLSs was associated with a higher risk of cancer recurrence and a poorer prognosis compared with intratumoral TLSs. However, in most cancers, there was no clear association between the location of TLSs and the outcome of treatment (6). Therefore, the location information of the TLSs was not refined for evaluation in this study.

Tumor-associated inflammatory responses have important effects on tumor development. Tumor-associated inflammation can lead to tumorigenesis by inducing genetic mutations, genomic instability, and epigenetic modifications that promote cancer cell proliferation and angiogenesis (23). Additionally, the systemic inflammatory response caused by the tumor accelerates a state of excessive nutritional depletion, which can increase cachexia. The results of this study revealed that low SII levels were associated with favorable DFS in patients with NSCLC who received neoadjuvant immune combination chemotherapy. There are various reasons for this outcome: Firstly, the rise in lymphocytes (CD4+ T cells, CD8+ T cells, et al.) enhances the cytotoxic immune response, which prevents the proliferation, invasion and migration of malignant cells (24). Secondly, the reduction in neutrophils hinders the secretion of pro-angiogenic, growth, and anti-apoptotic factors, thereby suppressing the growth and progression of cancer (25). Additionally, the decrease in platelets increases the susceptibility of circulating tumor cells to shear stress while in circulation, which impedes the initiation of epithelial-mesenchymal transition and mitigates the metastasis of cancer cells (26).

The inflammatory environment is closely associated with the formation and maturation of TLSs. Chronic persistent inflammation caused by tumors leads to the extranodal implantation of lymphoid tissue, which results in the formation of TLSs in the tumor and surrounding sites (27). The development of TLSs is a complicated process in which immune cell-derived pro-inflammatory signals are considered inducers. In this study, we found that a reduction in the inflammatory index PLR facilitated high TLS expression. This may be explained by the fact that the state of platelets and lymphocytes in NSCLC after neoadjuvant immune combination chemotherapy creates a specific inflammatory microenvironment that specifically influences TLSs formation and maturation. Therefore, this study incorporated both inflammatory environmental indicators that affect TLS formation, maturation, and expression into a prognostic model for NSCLC, which accurately predicted DFS durations in patients with NSCLC. More research is expected in the future to clarify the connection between the inflammatory environment of tumors and the anti-tumor immune response to find targets to enhance the effectiveness of immunotherapy.

In clinical practice, we can make a preliminary assessment of a patient’s immune therapy response and prognosis based on their inflammatory biomarkers. This assessment can then be combined with a comprehensive analysis of the patient’s immune scores and gene expression status to determine if they are suitable for neoadjuvant immune-chemo combination therapy. To our knowledge, this is the first study to investigate the relationship between inflammatory biomarkers with tumor microenvironment TLSs in patients with NSCLC and identify the easily assessable inflammatory index, PLR, as a reflection of TLS status. Changes TLS status, detected through inflammatory biomarkers in the blood, provides a basis for elucidating specific inflammatory microenvironments that promote TLS expression and offers novel insights into immuno-microenvironment research. Additionally, this study demonstrated that TLSs in the tumor microenvironment correlate not only with local factors but also with systemic immune conditions, providing evidence of a novel mechanism by which systemic inflammation aids in regulating tumor microenvironment immunity.

This study has some limitations. First, this was a single-center retrospective study with a small sample size, and the results may have been influenced by individual differences, the geographic area, or specific environmental factors. Second, the follow-up period of this study took place over a limited duration. Therefore, most of the tumor progression of the patients in this study was dominated by recurrence and metastasis, and fewer patients died, making it difficult to study the survival status of the patients. Third, only patients with resectable NSCLC who were treated with neoadjuvant immune combination chemotherapy were included in this study, and the presence of platinum-based and paclitaxel drugs or pemetrexed disodium interfered with and did not allow accurate assessment of the effect of PD-1 inhibitors on TLSs. The PD-1 checkpoint inhibitors and chemotherapeutic drugs used in the study may also have affected the patients’ prognosis because they were from different manufacturers. Additionally, the number of tissue sections observed for each patient was small and did not allow for a comprehensive assessment of TLS abundance and maturation in and around the entire tumor. Although the study showed a correlation between the inflammatory environment and TLSs, expect more basic experimental studies in the future to further explore their mechanisms.

In conclusion, our study indicated that low PLR was an independent predictor of TLS(+) and that both TLSs and SII predicted prognosis in patients with resectable NSCLC receiving neoadjuvant chemoimmunotherapy. Notably, the combination of SII and TLS to assess DFS duration in patients with NSCLC was more accurate than using either parameter alone.

## Data availability statement

The raw data supporting the conclusions of this article will be made available by the authors, without undue reservation.

## Ethics statement

Written informed consents were provided by the patients participants/participants. The study was carried out in accordance with the Helsinki Declaration and approved by the Ethics Review Committee of Shandong Cancer Hospital in China. (SDTHEC2022010006).

## Author contributions

Study design: SY, FX. Data acquisition and analysis HZ, FX. Interpretation of the data: FX, LL, NL. Drafting of the manuscript: FX. Revision of the manuscript: SY. All authors contributed to the article and approved the submitted version.
