# Supplementary material for: Combined inflammatory parameters and tertiary lymphoid structure predict prognosis in patients with resectable non-small cell lung cancer treated with neoadjuvant chemoimmunotherapy
Source: Front Immunol. 2023 Dec 14;14:1244256. doi: 10.3389/fimmu.2023.1244256 (PMC10752966; doi:10.3389/fimmu.2023.1244256)
Supplement: Supplementary file 1 [file Table_1.docx]

**Supplement Table 1** | Baseline characteristics of the enrolled patients

| Variables | No.(%) |
| --- | --- |
| **Age** |  |
| ＜63 | 58(49.6%) |
| ≥63 | 59(50.4%) |
| **Gender** |  |
| male | 96(82.1%) |
| female | 21(17.9%) |
| **Smoking history** |  |
| smoker or ex-smoker | 69(59.0%) |
| never smoker | 48(41.0%) |
| **Histology** |  |
| Squamous cell carcinoma | 71(60.7%) |
| Adenocarcinoma | 46(39.3%) |
| **ECOG PS** |  |
| 0 | 67(57.3%) |
| 1 | 39(33.3%) |
| ≥2 | 11(9.4%) |
| **Neoadjuvant therapy no of cycles** |  |
| 2 | 77(65.8%) |
| ＞2 | 40(34.2%) |
| **T Stage** |  |
| 1 | 8(6.8%) |
| 2 | 68(58.1%) |
| 3 | 22(18.8%) |
| 4 | 19(16.2%) |
| **N Stage** |  |
| 0 | 24(20.5%) |
| 1 | 25(21.4%) |
| 2 | 67(57.3%) |
| 3 | 1(0.1%) |
| **PD-L1** |  |
| Positive | 59(50.4%) |
| Negative | 23(19.7%) |
| Unknown | 35(29.9%) |
| **SII** |  |
| ≤822.63 | 72(61.5%) |
| ＞822.63 | 45(38.5%) |
| **NLR** |  |
| ≤3.59 | 74(63.2%) |
| ＞3.59 | 43(36.8%) |
| **PLR** |  |
| ≤288.78 | 98(83.8%) |
| ＞288.78 | 19(16.2%) |
| **LMR** |  |
| ≤3.04 | 55(47.0%) |
| ＞3.04 | 62(53.0%) |
| **TLS density** |  |
| 0 | 14(12.0%) |
| 1 | 38(32.5%) |
| 2 | 65(55.5%) |
| **TLS maturity** |  |
| with mature TLS | 77(65.8%) |
| no mature TLS | 40(34.2%) |
