# Supplementary material for: Combined inflammatory parameters and tertiary lymphoid structure predict prognosis in patients with resectable non-small cell lung cancer treated with neoadjuvant chemoimmunotherapy
Source: Front Immunol. 2023 Dec 14;14:1244256. doi: 10.3389/fimmu.2023.1244256 (PMC10752966; doi:10.3389/fimmu.2023.1244256)
Supplement: Supplementary file 2 [file Table_2.docx]

**Supplement Table 2 | Optimal cut-off values for inflammatory parameters**

| **Inflammatory indicators** | **AUC** | **Sensitivity** | **Specificity** | **95%CI** | **Cut-off point** |
| --- | --- | --- | --- | --- | --- |
| **SII** | 0.628 | 0.690 | 0.542 | 0.527-0.729 | 822.63 |
| **NLR** | 0.619 | 0.759 | 0.492 | 0.518-0.721 | 3.59 |
| **PLR** | 0.600 | 0.966 | 0.288 | 0.497-0.703 | 288.78 |
| **LMR** | 0.509 | 0.517 | 0.576 | 0.404-0.615 | 3.04 |

AUC=area under curve,CI=confidence interval.
